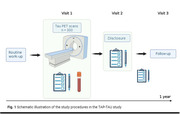# The impact of tau‐PET in a selected memory clinic cohort: design and rationale of the TAP‐TAU study

**DOI:** 10.1002/alz.091206

**Published:** 2025-01-09

**Authors:** Marie R. Vermeiren, Joost Somsen, Gert Luurtsema, Fransje E. Reesink, Nicolaas A. Verwey, Liesbeth Hempenius, Nelleke Tolboom, Geert Jan Biessels, J. Matthijs Biesbroek, Meike W. Vernooij, Sophie E.M. Veldhuijzen van Zanten, Harro Seelaar, Emma M. Coomans, Charlotte Teunissen, Afina Willemina Lemstra, Argonde C. van Harten, Leonie N.C. Visser, Wiesje M. van der Flier, Elsmarieke van de Giessen, Rik Ossenkoppele

**Affiliations:** ^1^ Alzheimer Center Amsterdam, Amsterdam UMC, Amsterdam Netherlands; ^2^ Department of Radiology & Nuclear Medicine, Amsterdam UMC, Amsterdam Netherlands; ^3^ Department of Nuclear Medicine and Molecular Imaging, University of Groningen, University Medical Center Groningen, Groningen Netherlands; ^4^ Department of Neurology, University of Groningen, University Medical Center Groningen, Groningen Netherlands; ^5^ Department of Neurology, Medical Center Leeuwarden, Leeuwarden Netherlands; ^6^ Geriatric Center, Medical Center Leeuwarden, Leeuwarden Netherlands; ^7^ Department of Radiology and Nuclear Medicine, University Medical Centre Utrecht, Utrecht Netherlands; ^8^ Department of Neurology and Neurosurgery, UMC Utrecht Brain Center, University Medical Center Utrecht, Utrecht Netherlands; ^9^ Department of Neurology, Diakonessenhuis Hospital, Utrecht Netherlands; ^10^ Department of Radiology and Nuclear Medicine, Erasmus University Medical Center, Rotterdam Netherlands; ^11^ Department of Neurology, Erasmus Medical Center, Rotterdam Netherlands; ^12^ Neurochemistry Laboratory, Amsterdam UMC, Amsterdam Netherlands; ^13^ Division of Clinical Geriatrics, Center for Alzheimer Research, Department of Neurobiology, Care Sciences and Society, Karolinska Institutet, Stockholm Sweden; ^14^ Department of Medical Psychology, Amsterdam UMC, University of Amsterdam, Amsterdam Netherlands; ^15^ Alzheimer Center Amsterdam, Neurology, Vrije Universiteit Amsterdam, Amsterdam UMC location VUmc, Amsterdam Netherlands; ^16^ Clinical Memory Research Unit, Lund University, Lund Sweden

## Abstract

**Background:**

Tau‐PET is a diagnostic tool with high sensitivity and high specificity for discriminating Alzheimer Disease (AD) dementia from other neurodegenerative disorders in well‐controlled research environments. The role of tau‐PET in “real‐world” clinical practice, however, remains to be established. We hypothesize that tau‐PET will lead to some changes of the pre‐PET clinical diagnosis and will improve diagnostic certainty and patient management in patients with considerable diagnostic uncertainty. The aim of the TAP‐TAU study is therefore to investigate the impact of tau‐PET in clinical practice.

**Method:**

The TAP‐TAU study, is a prospective, longitudinal multi‐center study in 300 patients (≥50 years old) with prodromal or mild dementia recruited in six Dutch memory clinics. Patients are eligible if diagnostic certainty is <85% after routine dementia screening and if the differential diagnosis includes AD. For example, patients i) are suspected of having mixed pathology (e.g., AD and vascular pathology), and/or ii) have an atypical clinical presentation, and/or iii) show conflicting or inconclusive outcomes on other biomarkers (e.g., magnetic resonance imaging or cerebrospinal fluid). Participants will undergo a [^18^F]flortaucipir tau‐PET scan, blood‐based biomarker sampling, and fill out patient wellbeing surveys (Figure 1). The primary outcome measures are change (pre‐ versus post‐ tau‐PET) in diagnosis, diagnostic certainty, patient management and patient wellbeing. Secondary outcome measures are head‐to‐head comparisons between tau‐PET and less‐invasive, lower cost and more scalable diagnostic tools such as novel blood‐based biomarkers and artificial intelligence based classifiers. A control group will be formed by patients who do not wish to undergo any study intervention (n=60). All participants will be followed for a year in the memory clinic.

**Result:**

The first participants will be enrolled in March 2024.

**Conclusion:**

In TAP‐TAU, we will investigate the added clinical value of tau‐PET in a real‐world clinical setting, including memory clinic patients with limited diagnostic certainty after routine work‐up. Findings of our study may contribute to recommendations regarding which patients may benefit most from assessment with tau‐PET. This study is timely in the dawning era of disease‐modifying treatments as vigilance concerning mixed pathologies and atypical presentations in the memory clinic population grows.